# Magnetic-Field-Orientation Dependent Thermal Entanglement of a Spin-1 Heisenberg Dimer: The Case Study of Dinuclear Nickel Complex with an Uniaxial Single-Ion Anisotropy

**DOI:** 10.3390/molecules26113420

**Published:** 2021-06-05

**Authors:** Azadeh Ghannadan, Jozef Strečka

**Affiliations:** Department of Theoretical Physics and Astrophysics, Faculty of Science, P. J. Šafárik University, Park Angelinum 9, 040 01 Košice, Slovakia; azadeh.ghannadan@student.upjs.sk

**Keywords:** spin-1 Heisenberg dimer, bipartite entanglement, exchange and single-ion anisotropy, dinuclear nickel complexes

## Abstract

The bipartite entanglement in pure and mixed states of a quantum spin-1 Heisenberg dimer with exchange and uniaxial single-ion anisotropies is quantified through the negativity in a presence of the external magnetic field. At zero temperature the negativity shows a marked stepwise dependence on a magnetic field with two abrupt jumps and plateaus, which can be attributed to the quantum antiferromagnetic and quantum ferrimagnetic ground states. The magnetic-field-driven phase transition between the quantum antiferromagnetic and quantum ferrimagnetic ground states manifests itself at nonzero temperatures by a local minimum of the negativity, which is followed by a peculiar field-induced rise of the negativity observable in a range of moderately strong magnetic fields. The rising temperature generally smears out abrupt jumps and plateaus of the negativity, which cannot be distinguished in the relevant dependencies above a certain temperature. It is shown that the thermal entanglement is most persistent against rising temperature at the magnetic field, for which an energy gap between a ground state and a first excited state is highest. Besides, temperature variations of the negativity of the spin-1 Heisenberg dimer with an easy-axis single-ion anisotropy may exhibit a singular point-kink, at which the negativity has discontinuity in its first derivative. The homodinuclear nickel complex [Ni${}_{2}$(Medpt)${}_{2}$(μ-ox)(H${}_{2}$O)${}_{2}$](ClO${}_{4}$)${}_{2}$·2H${}_{2}$O provides a suitable experimental platform of the antiferromagnetic spin-1 Heisenberg dimer, which allowed us to estimate a strength of the bipartite entanglement between two exchange-coupled Ni${}^{2+}$ magnetic ions on the grounds of the interaction constants reported previously from the fitting procedure of the magnetization data. It is verified that the negativity of this dinuclear compound is highly magnetic-field-orientation dependent due to presence of a relatively strong uniaxial single-ion anisotropy.

## 1. Introduction

Entanglement is one of the most peculiar features of quantum mechanics that does not have a classical counterpart and resulted a controversial debate between two prominent groups of physicists [1] in the 1930s. Protagonists of the so-called realist viewpoint represented by Einstein, Podolsky and Rosen considered quantum mechanics as an incomplete theory that has to be complemented by certain hidden variable(s) to avoid its indeterminism [2] in opposite to proponents of the orthodox viewpoint represented by Bohr and their followers [3]. This contradiction has been ultimately resolved only after Bell formulated famous inequalities, which proved that any local hidden-variable theory is incompatible with quantum mechanics [4]. It turned out that the principle of locality as the fundamental assumption of the physical realism prohibiting superluminal propagation of action on a distance must be rejected, because the violation of Bell inequalities has been decisively corroborated in numerous experiments requiring the repudiation of the locality principle [5].

The quantum entanglement as a primary source of instantaneous action on a distance (nonlocality) in quantum mechanics currently attracts renewed interest, because the entanglement seems to be indispensable for a development of novel quantum technologies, quantum computers and quantum information science [6,7]. The development of novel technologies based on fully quantum grounds is unavoidable at least for two principal reasons [8,9]. The first one closely relates to the miniaturization as the long-lasting trend in technological innovations. The devices built on constantly smaller scales will ultimately reach length scales of nanometers or angular momentum scales of Planck’s constant what necessarily means that their design must be based on quantum-mechanical principles [8]. The second reason lies in that devices exploiting quantum-mechanical principles can substantially outperform performance of devices based on classical grounds [9].

One of the most challenging current tasks in this rapidly developing research field is to find a suitable physical realization of the quantum computer, which would serve as a hardware for performing computational tasks with the help of efficient quantum algorithms [10,11,12]. Electron spin systems represent one of promising candidates for a design of quantum computers, because a two-level character of the electron spin provides the simplest platform to encode a quantum bit [13]. Of course, two well defined energy levels of the electron spin do not automatically guarantee implementation of a qubit owing to the fact that a quantum superposition of states is often extremely fragile against uncontrolled interactions of a qubit with its environment, i.e., the phenomenon referred to as a quantum decoherence. The loss of quantum information due to the decoherence is regarded as the most principal obstacle for the development of all quantum technologies exploiting solid-state materials [14].

Molecular-based magnetic materials, which are composed from weakly coupled discrete magnetic molecules, belong to the most perspective electron spin systems for a quantum computation [15,16,17] and quantum information processing [18,19]. The molecular magnets generally possess well defined pattern of discrete energy levels, whereas the associated quantum states can be easily tuned and coherently manipulated by the pulsed ESR technique [20]. The molecular magnetic materials thus naturally satisfy most important requirements imposed on basic building blocks of quantum computers [21,22] and hence, they can be regarded as prominent resources for the quantum computation [15,16,17], the storage and processing of quantum information [18,19]. Implementation of Grover’s search algorithm for instance requires a quantum superposition of ’single-molecule’ spin states, which may be addressed through a multi-frequency sequence of electromagnetic pulses according to the protocol developed by Leuenberger and Loss [15]. It turns out, moreover, that exchange-coupled magnetic molecules afford convenient resource for the implementation of quantum-mechanically entangled gates [23,24,25,26]. The quantum entanglement is eventually thought of as a key feature, which provides quantum algorithms an enormous advantage over classical algorithms. The molecular magnetic materials, which are composed of greater number of metal centers with nonzero resultant spin, may thus provide a route to large-scale quantum computations based on controlled logic gates employing the quantum entanglement between electron spins [27,28,29]. Shor’s factoring algorithm [12] as one of the most efficient quantum algorithms indeed addresses the computational challenge of factoring to prime numbers by exploiting the entanglement between ‘many-particle’ states.

From the theoretical point of view, the molecular-based magnetic materials are traditionally described by quantum Heisenberg spin models, which allow qualitative as well as quantitative characterization of their magnetic properties and quantum entanglement. The bipartite entanglement within pure and mixed states of the Heisenberg spin models can be for instance quantified in terms of von Neumann entropy of the reduced density matrix [30], concurrence [31,32] or negativity [33,34]. In addition, these entanglement measures can be related to measurable magnetic and thermodynamic quantities [35] and hence, they are also amenable to experimental testing [36,37]. While the concurrence and related entanglement of formation [31,32] is perhaps the most widely used entanglement measure for spin-1/2 Heisenberg systems constituted from two-dimensional qubits, the negativity is most commonly used entanglement measure for more general spin-*S* Heisenberg systems with higher spin magnitude S>1/2 providing platform built from *d*-dimensional qudits. In general, the entanglement features of the spin-*S* (S>1/2) Heisenberg systems are much less studied in comparison with their spin-1/2 counterparts owing to higher computational complexity [6,7].

As far as the spin-1 Heisenberg systems are concerned, the concept of negativity was applied in order to investigate a thermal entanglement of the spin-1 Heisenberg chain with different number of spins assuming bilinear and biquadratic interactions [38,39]. The thermal entanglement of a spin-1 Heisenberg dimer in an inhomogeneous magnetic field [40], Dzyaloshinskii–Moriya interaction [41] or under the concurrent effect of inhomogeneous magnetic field and Dzyaloshinskii–Moriya interaction [42] was also examined in particular. The relation between the quantum entanglement and quantum phase transitions of spin-1 Heisenberg clusters [43] and chain [44] were studied by an exact diagonalization and renormalization group method, respectively. Interestingly, the threshold temperature of a quantum spin-1 XY chain, at which the thermal entanglement vanishes, turns out to be independent of the number of spins [45]. The dimerization and spin frustration also have highly nontrivial effect upon the thermal entanglement of a spin-1 Heisenberg chain with an anisotropic exchange interaction [46]. The thermal entanglement of a spin-1 Heisenberg dimer with both linear and nonlinear coupling terms was studied on an optical lattice in presence of the magnetic field, which demonstrated that biquadratic interaction may enhance the thermal entanglement [47].

In the present paper, we will investigate in detail the quantum and thermal entanglement within pure and mixed states of a spin-1 Heisenberg dimer accounting for the exchange anisotropy, uniaxial single-ion anisotropy and magnetic field. The strength of bipartite entanglement of a spin-1 Heisenberg dimer will be examined in detail depending on temperature, magnetic field and uniaxial single-ion anisotropy. It is noteworthy that the homodinuclear nickel complex [Ni${}_{2}$(Medpt)${}_{2}$(µ-ox)(H${}_{2}$O)${}_{2}$](ClO${}_{4}$)${}_{2}$·2H${}_{2}$O (NAOC) [48] serves as an experimental realization of the investigated spin-1 Heisenberg dimer. We will also take advantage of the available magnetic data reported previously for the NAOC complex [49,50] and theoretical analysis of the respective coupling constants [51,52] in order to quantify a strength of the bipartite entanglement within this molecular-based magnetic material. In particular, we will clarify on this specific molecular magnetic material robustness of the thermal entanglement of the NAOC complex against temperature and magnetic field.

This paper is organized as follows. In Section 2, we will introduce a spin-1 Heisenberg model and obtain an exact solution for the negativity. In Section 3, we present typical dependencies of the negativity as a function of temperature and magnetic field for a few different values of uniaxial single-ion anisotropy. The implications for the thermal entanglement of the NAOC complex are presented in Section 4. Finally, the conclusions and summary of the most important findings are presented in Section 5. Some technical details of the calculation procedure are presented in Appendix A and Appendix B.

## 2. Model and Method

Let us consider the spin-1 Heisenberg dimer defined through the Hamiltonian:

(1) $$\hat{H}=J\left[\Delta \left({\hat{S}}_{1}^{x}{\hat{S}}_{2}^{x}+{\hat{S}}_{1}^{y}{\hat{S}}_{2}^{y}\right)+{\hat{S}}_{1}^{z}{\hat{S}}_{2}^{z}\right]+D\left[{\left({\hat{S}}_{1}^{z}\right)}^{2}+{\left({\hat{S}}_{2}^{z}\right)}^{2}\right]-h\left({\hat{S}}_{1}^{z}+{\hat{S}}_{2}^{z}\right),$$

where ${\hat{S}}_{j}^{\alpha }$ (α=x,y,z) are three spatial components of the spin-1 operator assigned to two different magnetic ions numbered by the suffix j=1 and 2. The coupling constant *J* determines a size of the XXZ exchange interaction, the parameter Δ stands for the spatial anisotropy in this exchange interaction, and the parameter *D* is the uniaxial single-ion anisotropy. Finally, the Zeeman’s term $h=g\mu_{B}B$ accounts for the effect of the external magnetic field *B* ($\mu_{B}$ is the Bohr magneton and *g* is the Landé *g*-factor). The energy eigenvalues, eigenvectors and basic magnetic properties of the spin-1 Heisenberg dimer given by the Hamiltonian (1) were exactly calculated and comprehensively discussed in reference [51], to which readers interested in further details are referred to. For completeness, the eigenvalues and eigenvectors of the Hamiltonian (1) are listed in Appendix A together with the explicit form of the relevant partition function.

To quantify a degree of the bipartite entanglement, one may take advantage of several entanglement measures [6], whereas the concurrence and negativity are eventually the most common ones. The concurrence is usually used as a measure of the bipartite entanglement for the spin-1/2 qubits, while the negativity is more general quantity that may be straightforwardly used as a measure of the bipartite entanglement also for spin-1 qutrits. According to the Peres–Horodecki separability criterion [33], the negativity becomes non-zero just if the state is inseparable and hence, it may be indeed used as an indicator of the bipartite entanglement. To calculate the negativity of the spin-1 Heisenberg dimer (1) one has first to calculate a density operator using the relation:

(2) $$\hat{\rho }=\frac{1}{Z}\exp\left(-\beta \hat{H}\right)=\frac{1}{Z}\sum_{i=1}^{9}\exp\left(-\beta E_{i}\right)|\psi_{i}\rangle \langle \psi_{i}|,$$

where $\beta =1/(k_{B}T)$, $k_{B}$ is Boltzmann’s constant, *T* is the absolute temperature, $E_{i}$ and $|\psi_{i}\rangle$ are the respective eigenenergies and eigenvectors of the Hamiltonian (1) obtained by solving the relevant eigenvalue problem $\hat{H}|\psi_{i}\rangle =E_{i}|\psi_{i}\rangle$ and Z is the partition function $Z=\sum_{i=1}^{9}\exp\left(-\beta E_{i}\right)$. It is worthwhile to recall that the complete set of eigenenergies $E_{i}$ and eigenvectors $|\psi_{i}\rangle$ was exactly calculated in our previous work [51], so we have decided to quote in Appendix A just the relevant final results in order to make the present paper self-contained. After taking into consideration the eigenvectors of the Hamiltonian (1) explicitly listed in Appendix A, the density matrix $\rho_{ij}=\langle S_{1}^{z}{}^{\prime },S_{2}^{z}{}^{\prime }|\hat{\rho }|S_{1}^{z},S_{2}^{z}\rangle$ acquires in the standard basis $|S_{1}^{z},S_{2}^{z}\rangle$ spanned over the eigenfunctions of *z*-components of both spins the following matrix representation:

(3) $$\rho_{ij}=\begin{array}{cc} \; & \begin{array}{ccccccccc} |1,1\rangle & |1,0\rangle & |1,-1\rangle & |0,1\rangle & |0,0\rangle & |0,-1\rangle & |-1,1\rangle & |-1,0\rangle & |-1,-1\rangle \end{array}\\ \begin{array}{c} \begin{array}{c} \langle 1,1| \end{array}\\ \langle 1,0|\\ \langle 1,-1|\\ \langle 0,1|\\ \langle 0,0|\\ \langle 0,-1|\\ \langle -1,1|\\ \langle -1,0|\\ \langle -1,-1| \end{array} & \left(\begin{array}{ccccccccc} \rho_{11} & 0 & 0 & 0 & 0 & 0 & 0 & 0 & 0\\ 0 & \rho_{22} & 0 & \rho_{24} & 0 & 0 & 0 & 0 & 0\\ 0 & 0 & \rho_{33} & 0 & \rho_{35} & 0 & \rho_{37} & 0 & 0\\ 0 & \rho_{42} & 0 & \rho_{44} & 0 & 0 & 0 & 0 & 0\\ 0 & 0 & \rho_{53} & 0 & \rho_{55} & 0 & \rho_{57} & 0 & 0\\ 0 & 0 & 0 & 0 & 0 & \rho_{66} & 0 & \rho_{68} & 0\\ 0 & 0 & \rho_{73} & 0 & \rho_{75} & 0 & \rho_{77} & 0 & 0\\ 0 & 0 & 0 & 0 & 0 & \rho_{86} & 0 & \rho_{88} & 0\\ 0 & 0 & 0 & 0 & 0 & 0 & 0 & 0 & \rho_{99} \end{array}\right) \end{array}.$$

For the sake of brevity, all nonzero elements of the density matrix (3) are specifically quoted in Appendix B. The negativity N can be consequently calculated from negative eigenvalues $\lambda_{i}<0$ of a partially transposed density matrix according to the formula derived by Vidal and Werner [34]:

(4) $$N=\sum_{\lambda_{i}<0}\left|\lambda_{i}\right|.$$

The partial transposition means that the states of one subsystem are kept, while the states of other subsystem are interchanged. If the states of the first spin are kept and a partial transposition is made with respect to the states of the second spin, then, one gets the following matrix representation of a partially transposed density matrix $\rho^{T_{2}}=\langle S_{1}^{z}{}^{\prime },S_{2}^{z}{}^{\prime }|\hat{\rho }|S_{1}^{z},S_{2}^{z}\rangle^{T_{2}}=\langle S_{1}^{z}{}^{\prime },S_{2}^{z}|\hat{\rho }|S_{1}^{z},S_{2}^{z}{}^{\prime }\rangle$:

(5) $$\rho^{T_{2}}=(\begin{array}{c} \begin{array}{ccccccccc} \rho_{11} & 0 & 0 & 0 & \rho_{24} & 0 & 0 & 0 & \rho_{37}\\ 0 & \rho_{22} & 0 & 0 & 0 & \rho_{35} & 0 & 0 & 0\\ 0 & 0 & \rho_{33} & 0 & 0 & 0 & 0 & 0 & 0\\ 0 & 0 & 0 & \rho_{44} & 0 & 0 & 0 & \rho_{57} & 0\\ \rho_{24} & 0 & 0 & 0 & \rho_{55} & 0 & 0 & 0 & \rho_{68}\\ 0 & \rho_{35} & 0 & 0 & 0 & \rho_{66} & 0 & 0 & 0\\ 0 & 0 & 0 & 0 & 0 & 0 & \rho_{77} & 0 & 0\\ 0 & 0 & 0 & \rho_{57} & 0 & 0 & 0 & \rho_{88} & 0\\ \rho_{37} & 0 & 0 & 0 & \rho_{68} & 0 & 0 & 0 & \rho_{99} \end{array} \end{array}).$$

The block-diagonal form of the partially transposed density matrix $\rho^{T_{2}}$ given by Equation (5) allows a straightforward calculation of all its eigenvalues:

(6) $$\begin{array}{c} \begin{array}{cc} \lambda_{1} & =\rho_{33},\\ \lambda_{2} & =\rho_{77},\\ \lambda_{3} & =\frac{1}{2}\left[\rho_{22}+\rho_{66}+\sqrt{{\left(\rho_{22}-\rho_{66}\right)}^{2}+4\rho_{35}^{2}}\right],\\ \lambda_{4} & =\frac{1}{2}\left[\rho_{44}+\rho_{88}+\sqrt{{\left(\rho_{44}-\rho_{88}\right)}^{2}+4\rho_{57}^{2}}\right],\\ \lambda_{5} & =\frac{1}{2}\left[\rho_{22}+\rho_{66}-\sqrt{{\left(\rho_{22}-\rho_{66}\right)}^{2}+4\rho_{35}^{2}}\right],\\ \lambda_{6} & =\frac{1}{2}\left[\rho_{44}+\rho_{88}-\sqrt{{\left(\rho_{44}-\rho_{88}\right)}^{2}+4\rho_{57}^{2}}\right],\\ \lambda_{n} & =-\frac{a}{3}+2sgn\left(q\right)\cos\left[\frac{\phi +2\pi (n-7)}{3}\right],n=7,8,9. \end{array} \end{array}$$

The last three eigenvalues are expressed in terms of parameters *p*, *q* and ϕ:

(7) $$\begin{array}{ccc} p & = & \frac{a^{2}}{9}-\frac{b}{3},\\ q & = & -{\left(\frac{a}{3}\right)}^{3}+\frac{ab}{6}-\frac{c}{2},\\ \phi & = & \arctan\left(\frac{\sqrt{p^{3}-q^{2}}}{q}\right), \end{array}$$

which follow from the solution of a characteristic cubic equation $\lambda^{3}+a\lambda^{2}+b\lambda +c=0$ defined through the coefficients:

(8) $$\begin{array}{ccc} a & = & -(\rho_{11}+\rho_{55}+\rho_{99}),\\ b & = & \rho_{11}\rho_{55}+\rho_{55}\rho_{99}+\rho_{11}\rho_{99}-\left(\rho_{24}^{2}+\rho_{37}^{2}+\rho_{68}^{2}\right),\\ c & = & \rho_{11}\rho_{68}^{2}+\rho_{55}\rho_{37}^{2}+\rho_{99}\rho_{24}^{2}-\rho_{11}\rho_{55}\rho_{99}-2\rho_{24}\rho_{37}\rho_{68}. \end{array}$$

It is quite obvious that the eigenvalues $\lambda_{1}$, $\lambda_{2}$, $\lambda_{3}$, $\lambda_{4}$ are always positive and the detailed numerical analysis reveals the same feature also for the eigenvalue $\lambda_{9}$. The five positive eigenvalues of the partially transposed density matrix (5) do not contribute to the negativity (2), which is entirely determined by the remaining four eigenvalues that may become under certain conditions negative. The negativity of the spin-1 Heisenberg dimer can be accordingly calculated from the formula:

(9) $$N=\sum_{\lambda_{i}<0}\left|\lambda_{i}\right|=\sum_{i=5}^{8}\frac{|\lambda_{i}|-\lambda_{i}}{2}.$$

## 3. Theoretical Results and Discussion

Before proceeding to a detailed investigation of the negativity it is worthwhile to recall that the antiferromagnetic spin-1 Heisenberg dimer has according to reference [51] three different ground states denoted as the quantum antiferromagnetic phase $|\mathrm{QAF}\rangle$:

(10) $$|\mathrm{QAF}\rangle =\frac{1}{2}\left[A_{+}\left(|1,-1\rangle +|-1,1\rangle \right)-\sqrt{2}A_{-}|0,0\rangle \right],A_{\pm }=\sqrt{1\pm \frac{\frac{1}{2}-\frac{D}{J}}{\sqrt{{\left(\frac{1}{2}-\frac{D}{J}\right)}^{2}+2\Delta^{2}}}},$$

the quantum ferrimagnetic phase $|\mathrm{QFI}\rangle$:

(11) $$|\mathrm{QFI}\rangle =\frac{1}{\sqrt{2}}\left(|1,0\rangle -|0,1\rangle \right),$$

and the classical ferromagnetic phase $|\mathrm{FM}\rangle$:

(12) $$|\mathrm{FM}\rangle =|1,1\rangle .$$

**Figure 1:**
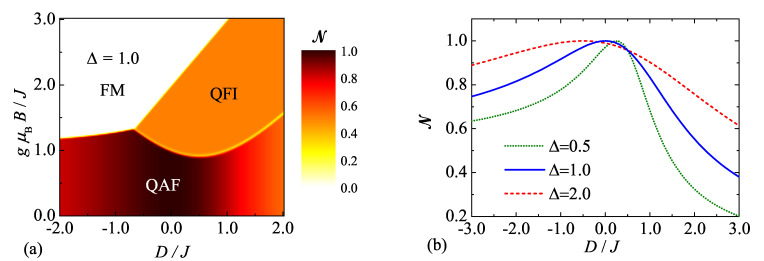
(**a**) Zero-temperature density plot of the negativity in D/J—$g\mu_{B}B/J$ plane for the isotropic coupling constant with Δ=1; (**b**) The negativity as a function of the uniaxial single-ion anisotropy D/J for three different values of the exchange anisotropy Δ=0.5,1.0 and 2.0 at zero temperature and zero magnetic field.

Zero-temperature density plot of the negativity, which is shown in Figure 1a in the plane uniaxial single-ion anisotropy versus magnetic field for the particular case with the isotropic exchange interaction Δ=1, is actually in a perfect agreement with the ground-state phase diagram reported previously in reference [51]. It is evident that the strongest quantum entanglement can be detected at low enough magnetic fields, where the quantum antiferromagnetic state $|\mathrm{QAF}\rangle$ constitutes the relevant ground state. It turns out that the negativity acquires its highest possible value N=1 within the $|\mathrm{QAF}\rangle$ ground state for the fully isotropic case with Δ=1 and D/J=0, whereas the uniaxial single-ion anisotropy of either easy-axis (D<0) or easy-plane (D>0) type suppresses a strength of the quantum entanglement. The quantum ferrimagnetic phase $|\mathrm{QFI}\rangle$ represents another available ground state emergent at moderately high magnetic fields and specific values of the uniaxial single-ion anisotropy D/J>−2/3, whereas the negativity equals to a half of its maximal value N=0.5 within this quantum ground state. Finally, the classical ferromagnetic phase $|\mathrm{FM}\rangle$ with zero negativity emerges as the last available ground state at sufficiently high magnetic fields independently of the uniaxial single-ion anisotropy. It is worthwhile to remark, moreover, that the negativity exhibits a discontinuous jump at any phase boundary between the $|\mathrm{QAF}\rangle$, $|\mathrm{QFI}\rangle$, and $|\mathrm{FM}\rangle$ ground states.

To bring a deeper insight into a concurrent effect of the exchange and uniaxial single-ion anisotropy upon a quantum entanglement, zero-temperature dependencies of the negativity are depicted in Figure 1b as a function on the uniaxial single-ion anisotropy D/J for zero magnetic field and three selected values of the exchange anisotropy Δ=0.5,1.0 and 2.0. Note that the quantum antiferromagnetic phase $|\mathrm{QAF}\rangle$ given by the eigenvector (10) represents the unique ground state emergent at zero magnetic field and hence, zero-temperature asymptotic value of the negativity follows from the density operator $\hat{\rho }=|\mathrm{QAF}\rangle \langle \mathrm{QAF}|$ having character of the projection operator for the respective ground state:

(13) $$N_{\mathrm{QAF}}=\frac{1}{4}\left(1+\frac{\frac{1}{2}-\frac{D}{J}+4\Delta }{\sqrt{{\left(\frac{1}{2}-\frac{D}{J}\right)}^{2}+2\Delta^{2}}}\right).$$

It is evident from the formula (13) that the negativity is within the $|\mathrm{QAF}\rangle$ ground state independent of the magnetic field and its maximum value N=1 is reached just for special combinations of the exchange and uniaxial single-ion anisotropies. While the easy-axis exchange anisotropy Δ<1 (e.g., Δ=0.5) shifts the global maximum of the negativity N=1 towards the easy-plane single-ion anisotropy D/J>0, the easy-plane exchange anisotropy Δ>1 (e.g., Δ=2.0) contrarily shifts this maximum towards the easy-axis single-ion anisotropy D/J<0. It could be thus concluded that the antiferromagnetic spin-1 Heisenberg dimer exhibits the strongest quantum entanglement, i.e., the highest possible value of the negativity N=1, either for the fully isotropic case with Δ=1,D/J=0 or when the easy-axis exchange anisotropy compensates the effect of easy-plane single-ion anisotropy or vice versa. Since the negativity displays qualitatively the same dependencies regardless of the exchange anisotropy Δ, from here onward we will focus our further attention to the most common particular case with the isotropic exchange interaction Δ=1.

**Figure 2:**
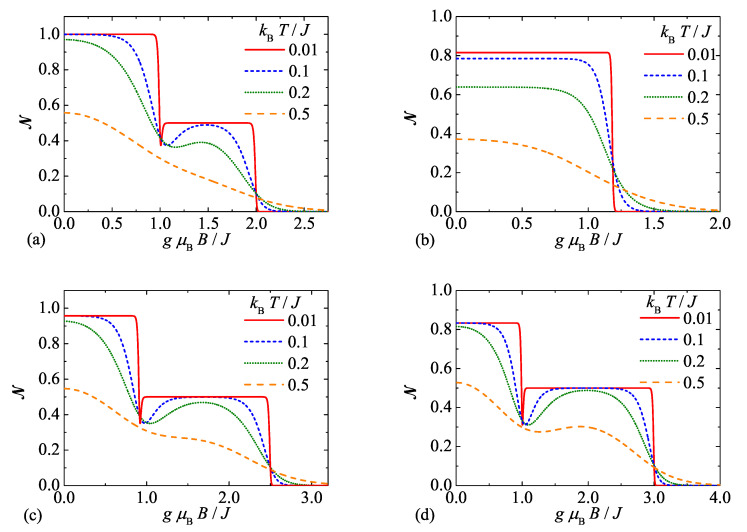
The negativity of the spin-1 Heisenberg dimer as a function of the magnetic field for the particular case with the isotropic exchange coupling Δ=1, a few selected values of temperature (see legend) and four different values of the uniaxial single-ion anisotropy: (**a**) D/J=0.0; (**b**) D/J=−2.0; (**c**) D/J=0.5; (**d**) D/J=1.0.

**Figure 3:**
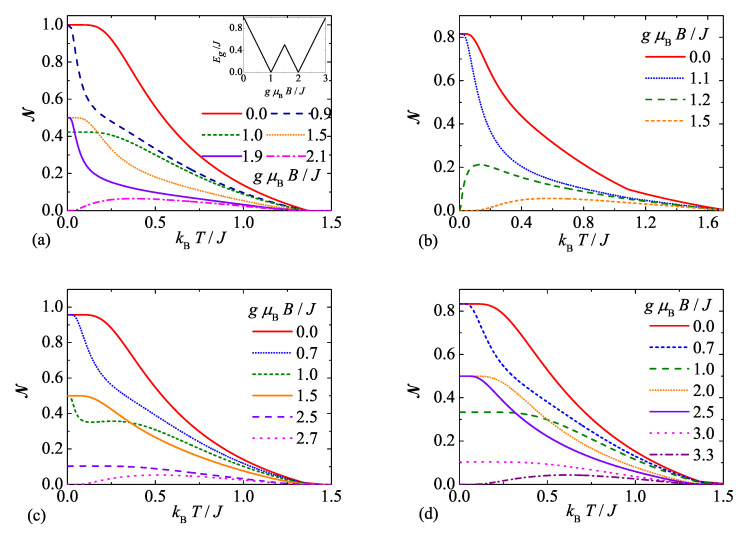
Temperature variations of the negativity of the spin-1 Heisenberg dimer for the particular case with the isotropic exchange coupling Δ=1, a few selected values of the magnetic field (see legend) and four different values of the uniaxial single-ion anisotropy: (**a**) D/J=0.0; (**b**) D/J=−2.0; (**c**) D/J=0.5; (**d**) D/J=1.0.

Figure 2 displays the negativity of the spin-1 Heisenberg dimer as a function of the external magnetic field for a few different temperatures and four selected values of the uniaxial single-ion anisotropy. Interestingly, the plateaus in the magnetic-field dependence of the negativity observable at low enough temperatures are quite reminiscent of the previously reported magnetization plateaus (confront Figure 2 with Figure 3 in reference [51]). However, this comparison also reveals an inverse relation between a size of the magnetization and negativity: the greater is the magnetization, the smaller is the negativity and the reverse statement also holds true. Two nonzero plateaus of the negativity are evident for the particular case without the single-ion anisotropy term D/J=0 (see Figure 2): the plateau at $N_{\mathrm{QAF}}=1$ corresponds to the quantum antiferromagnetic phase $|\mathrm{QAF}\rangle$ and the other one at $N_{\mathrm{QFI}}$ = 0.5 to the quantum ferrimagnetic phase $|\mathrm{QFI}\rangle$ while a trivial zero plateau $N_{\mathrm{FM}}$ = 0 corresponds to the classical ferromagnetic phase $|\mathrm{FM}\rangle$. The qualitatively same trends can be also observed whenever the uniaxial single-ion anisotropy satisfies the inequality $D/J>-\frac{2}{3}$ (see Figure 2c,d) except that the asymptotic value of the negativity at low magnetic fields is suppressed from its maximum value in accordance with the formula (13) derived for the $|\mathrm{QAF}\rangle$ ground state. Another interesting observation is that the negativity exhibits a peculiar nonmonotonic magnetic-field dependence in a vicinity of the transition field between the $|\mathrm{QAF}\rangle$ and $|\mathrm{QFI}\rangle$ ground states when it transiently drops down to a local minimum before it tends back to zero-temperature asymptotic value $N_{\mathrm{QFI}}$ = 0.5 of the latter ground state, whereas the width of this local minimum gradually diminishes upon lowering of the temperature. On the other hand, there exists just a single nontrivial plateau of the negativity without any nonmonotonic dependence or local minimum whenever one considers a sufficiently strong uniaxial single-ion anisotropy of easy-axis type $D/J<-\frac{2}{3}$, because the quantum antiferromagnetic ground state $|\mathrm{QAF}\rangle$ directly changes to the classical ferromagnetic one $|\mathrm{FM}\rangle$ upon increasing of the magnetic field (see Figure 2b). The magnetic fields, at which all abrupt changes of the negativity are detected at low enough temperatures, are all consistent with the ground-state phase diagram and transition fields reported in our previous study [51] (see also Figure 1a). It is also noteworthy that rising temperature evidently makes sharp stepwise dependencies of the negativity observable at low enough temperatures smoother, because the negativity gradually smears out due to higher thermal population of excited states within the relevant mixed states of the spin-1 Heisenberg dimer. Last but not least, it should be pointed out that the saturation field to the classical ferromagnetic phase $|\mathrm{FM}\rangle$ monotonically increases upon strengthening of the uniaxial single-ion anisotropy, which thus reinforces a resistance of the bipartite entanglement against the magnetic field.

Furthermore, typical temperature dependencies of the negativity of the spin-1 Heisenberg dimer are plotted in Figure 3 for a few selected values of the external magnetic field and four different values of the uniaxial single-ion anisotropy. As could be expected, the negativity mostly monotonically decreases upon increasing of temperature until it completely vanishes at a threshold temperature even though one may occasionally detect a more striking nonmonotonic temperature dependencies of the negativity. It is also worth mentioning that zero-temperature asymptotic values of the negativity are in accordance with the specific values $N_{\mathrm{QFI}}$ = 0.5 and $N_{\mathrm{QAF}}$ given by Equation (13), which were previously ascribed to the quantum ferrimagnetic phase $|\mathrm{QFI}\rangle$ and the quantum antiferromagnetic phase $|\mathrm{QAF}\rangle$, respectively. If the magnetic field is selected slightly above the saturation value, the negativity starts from zero in agreement with the classical character of the fully polarized ferromagnetic phase $|\mathrm{FM}\rangle$, then it steadily rises to a local maximum before it finally tends to zero at some threshold temperature. To bring insight into how robust is the quantum entanglement with respect to temperature within the quantum antiferromagnetic $|\mathrm{QAF}\rangle$ and ferrimagnetic $|\mathrm{QFI}\rangle$ phases, the inset of Figure 3a shows an energy gap between the ground state and first excited state. It is quite obvious that an energy gap above the $|\mathrm{QAF}\rangle$ ground state is the highest at zero magnetic field and so it is also the thermal dependence of the negativity. Similarly, the highest energy gap above the $|\mathrm{QFI}\rangle$ ground state coincides with the magnetic field $g\mu_{B}B/J=1.5$, at which the most robust thermal dependence of the negativity with the starting asymptotic value N=0.5 can be found. It could be thus concluded that the thermal stability of the quantum entanglement is proportional to an energy gap between the ground and first excited state.

**Figure 4:**
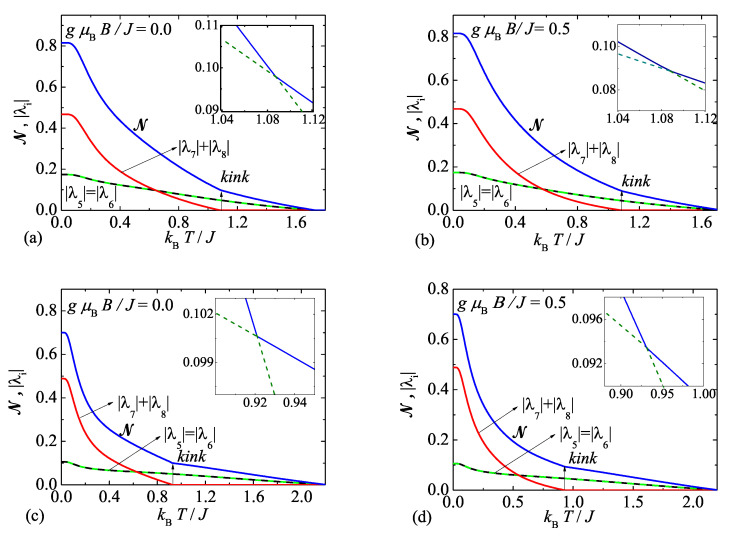
(**a**,**b**) Temperature dependencies of the overall negativity N and its three individual contributions $N_{1}$, $N_{2}$, and $N_{3}$ for the spin-1 Heisenberg dimer with the isotropic exchange coupling Δ=1, the uniaxial single-ion anisotropy D/J=−2.0 and two different magnetic fields: (**a**) B=0; (b) $g\mu_{B}B/J=0.5$; (**c**,**d**) The same as in the panel (**a**,**b**) just for the uniaxial single-ion anisotropy D/J=−4.0. The insets show a kink of the negativity in an enhanced scale.

By inspection we have found that the negativity is composed from three terms: $N_{1}=|\lambda_{7}\left|+\right|\lambda_{8}|$, $N_{2}=\left|\lambda_{5}\right|$, and $N_{3}=\left|\lambda_{6}\right|$, which relate to four eigenvalues (6) of the partially transposed density matrix (5) that may become under certain circumstances negative. It is noteworthy that the first term $N_{1}=|\lambda_{7}\left|+\right|\lambda_{8}|$ is the sum of two possibly negative eigenvalues of the partially transposed density matrix (5), which come from the solution of the characteristic cubic equation, whereas the eigenvalue $\lambda_{8}$ represents the analytical continuation of the eigenvalue $\lambda_{7}$ at higher temperatures. The individual contributions to the overall negativity of the antiferromagnetic spin-1 Heisenberg dimer are plotted in Figure 4 for two different values of the uniaxial single-ion anisotropy of the easy-axis type D/J=−2.0 and −4.0. It is found that the sum of eigenvalues $N_{1}=|\lambda_{7}\left|+\right|\lambda_{8}|$ provides the most dominant contribution to the negativity at sufficiently low temperatures, but this contribution simultaneously shows a steeper decline upon increasing of temperature in comparison with two negative eigenvalues $N_{2,3}=|\lambda_{5}\left|=\right|\lambda_{6}|$ contributing equally to the negativity. As a result, the negativity exhibits a remarkable kink at the specific temperature (e.g., $k_{B}T/J\approx 1.08$ for D/J=−2.0), which relates to a gradual breakdown of the first contribution $N_{1}=|\lambda_{7}\left|+\right|\lambda_{8}|$ and the first derivative of the negativity consequently displays discontinuity at the respective kink. Note furthermore that the negativity displays this striking kink even if one considers moderate values of the magnetic field $g\mu_{B}B/J=0.5$ (see Figure 4b), which gradually disappears from a thermal dependence of the negativity only at sufficiently high magnetic fields. It is worth mentioning that the kink is detectable in the thermal dependence of the negativity just for the uniaxial single-ion anisotropy of the easy-axis type as exemplified in Figure 4 on two particular cases with D/J=−2.0 and −4.0.

**Figure 5:**
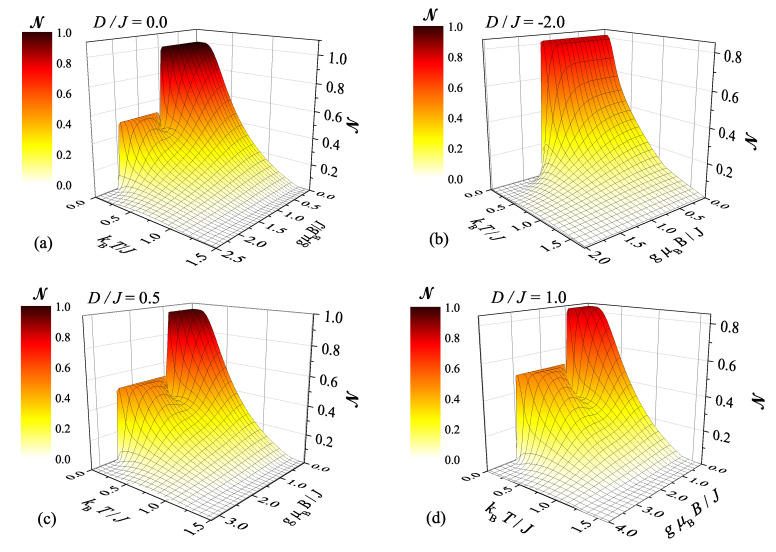
3D plots of the negativity of the spin-1 Heisenberg dimer with the isotropic exchange interaction Δ=1 as a function of temperature and magnetic field for four different values of the uniaxial single-ion anisotropy: (**a**) D/J=0.0; (**b**) D/J=−2.0; (**c**) D/J=0.5; (**d**) D/J=1.0.

To gain an overall insight, 3D plot of the negativity is depicted in Figure 5 as a function of temperature and magnetic field for four different values of the uniaxial single-ion anisotropy. The displayed plots nicely demonstrate all general features discussed previously and moreover, they also clearly allocate the parameter space with nonzero thermal entanglement. It is quite apparent from the relevant 3D plots that the rising temperature and magnetic field mostly suppress the negativity in accordance with common expectations, however, the negativity may also display an outstanding contraintuitive rise in a restricted range of temperatures and magnetic fields being sufficiently close to phase boundaries between two different ground states. Moreover, it turns out that the threshold temperature, above which the thermal entanglement (negativity) vanishes, is independent of the magnetic field for selected values of the exchange and uniaxial single-ion anisotropy. Last but not least, it also follows from Figure 5 that a weak thermal entanglement can be thermally induced above the classical $|\mathrm{FM}\rangle$ ground state, which is surprisingly stable against thermal attenuation regardless of its negligible magnitude.

## 4. Entanglement in the Dinuclear Nickel Complex NAOC

**Figure 6:**
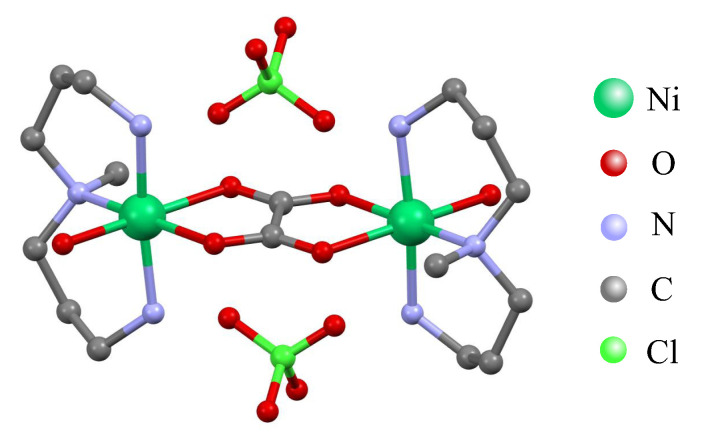
A crystal structure of the dinuclear nickel complex [Ni${}_{2}$(Medpt)${}_{2}$(μ-ox)(H${}_{2}$O)${}_{2}$](ClO${}_{4}$)${}_{2}$·2H${}_{2}$O abbreviated as NAOC (ox = oxalate and Medpt = 3,3${}^{\prime }$-diamino-N-methyl-dipropylamine) adapted according to crystallographic data reported in reference [48]. A color scheme for the atom labeling is shown in the legend, whereas hydrogen atoms are not shown for clarity.

In this part we will investigate in detail a bipartite entanglement between two exchange-coupled spin-1 Ni^2+^ magnetic ions of the homodinuclear coordination compound [Ni_2_(Medpt)_2_(μ-ox)(H_2_O)_2_](ClO_4_)_2_·2H_2_O (ox = oxalate and Medpt = 3,3′-diamino-N-methyl-dipropylamine) [48] referred to as the NAOC complex. Before doing so, a few comments are in order concerning with magneto-correlations of the NAOC complex whose crystal structure is displayed in Figure 6. First of all, it should be pointed out that the NAOC complex represents an excellent experimental realization of a spin-1 Heisenberg dimer, because two spin-1 Ni^2+^ magnetic ions are strongly coupled through superexchange pathways mediated by the bridging oxalate group and rather bulky tridentate blocking ligand Medpt makes intermolecular interactions negligible with respect to this dominant magnetic interaction. Besides, the highly distorted octahedral arrangements of ligands around each central Ni^2+^ magnetic ion indicates a substantial single-ion anisotropy. As a matter of fact, two amine groups of the tridentate ligand Medpt from axial positions of an octahedral coordination sphere are much closer to the central Ni^2+^ magnetic ion than other four ligands from its equatorial plane [48].

High-field magnetization data measured along two principal crystallographic $c^{*}$- and *a*-axes of a single-crystal sample of the NAOC complex [49,50] actually verify highly anisotropic magnetization process: the saturation magnetization is reached at much lower magnetic field if the external magnetic field is applied along the easy magnetization axis identified with the crystallographic $c^{*}$-axis in comparison with the hard magnetization axis identified with the crystallographic *a*-axis [49,50]. The magnetization curve of the NAOC complex along the crystallographic $c^{*}$-axis, which can be regarded as the principal quantization *z*-axis in a spin space, can be satisfactorily modeled by the spin-1 Heisenberg dimer (1) with the isotropic coupling constant $J/k_{B}=30.66$ K and Δ=1, the easy-axis uniaxial single-ion anisotropy $D_{c^{*}}/k_{B}=-12.48$ K and the gyromagnetic factor $g_{c^{*}}=2.28$[51]. On the other hand, the theoretical modeling of the magnetization curve of the NAOC complex along the crystallographic *a*-axis is much more complex due to off-diagonal character of the applied (transverse) magnetic field [52]. However, the spin-1 Heisenberg dimer (1) with the same coupling constant $J/k_{B}=30.66$ K and Δ=1, the hard-axis uniaxial single-ion anisotropy $D_{a}/k_{B}=4.91$ K and the gyromagnetic factor $g_{a}=2.24$[51] provides relatively plausible fit of the experimental data in spite of the fact the crystallographic *a*-axis is not true quantization axis. For completeness, it is worth noticing that the NAOC complex has a small biaxial single-ion anisotropy originating from the heterogeneity of ligands in an equatorial plane of the octahedral coordination sphere of the central spin-1 Ni${}^{2+}$ magnetic ions, which will be neglected for simplicity [52].

**Figure 7:**
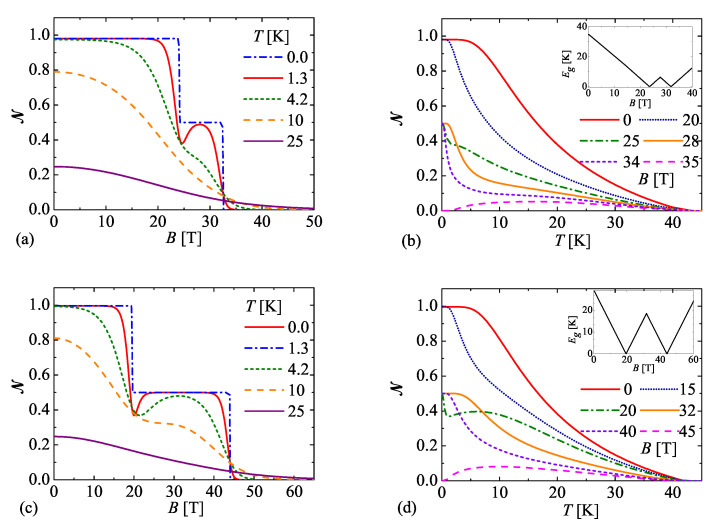
(**a**,**b**) The negativity of the NAOC complex when the external magnetic field is applied along the crystallographic $c^{*}$-axis. Magnetic-field [panel (**a**)] and temperature [panel (**b**)] dependencies of the negativity are based on the spin-1 Heisenberg dimer (1) with the isotropic coupling constant $J/k_{B}=30.66$ K and Δ=1, the uniaxial single-ion anisotropy $D_{c^{*}}/k_{B}=-12.48$ K and the gyromagnetic factor $g_{c^{*}}=2.28$; (**c**,**d**) The negativity of the NAOC complex when the external magnetic field is applied along the crystallographic *a*-axis. Magnetic-field [panel (**c**)] and temperature [panel (**d**)] dependencies of the negativity are based on the spin-1 Heisenberg dimer (1) with the isotropic coupling constant $J/k_{B}=30.66$ K and Δ=1, the uniaxial single-ion anisotropy $D_{a}/k_{B}=4.91$ K and the gyromagnetic factor $g_{a}=2.24$.

Now, let us adapt both reported fitting sets of the interaction parameters for the magnetization data measured along the crystallographic $c^{*}$- and *a*-axes in order to clarify the effect of magnetic field and temperature upon a thermal entanglement between two exchange-coupled spin-1 Ni${}^{2+}$ magnetic ions forming the dinuclear core of the NAOC complex. Figure 7a shows how the negativity of the NAOC complex depends on the external magnetic field oriented along the crystallographic $c^{*}$-axis being the easy magnetization axis. At zero temperature the negativity almost equals to its maximum value $N_{\mathrm{QAF}}\approx 1$ from zero field up to approximately 23 T due to presence of the quantum antiferromagnetic phase $|\mathrm{QAF}\rangle$, then it exhibits a rather narrow plateau exactly at a half of its maximum value $N_{\mathrm{QFI}}=0.5$ due to the quantum ferrimagnetic phase $|\mathrm{QFI}\rangle$ emergent in the field range from 23 T up to 34 T before it finally jumps to zero at the saturation field 34 T due to presence of the classical ferromagnetic phase $|\mathrm{FM}\rangle$. The stepwise changes of the negativity are of course gradually smeared out upon increasing of temperature, whereas strict jumps of the negativity appearing at zero temperature are replaced with a steep but continuous changes at small enough temperatures. However, the distinct profile of the negativity with two marked plateaus can be still clearly distinguished at small enough temperatures as for instance T=1.3 K used in the previous magnetization experiments [49,50]. Moreover, the field-driven phase transition between the $|\mathrm{QAF}\rangle$ and $|\mathrm{QFI}\rangle$ ground states is also clearly manifested at low enough temperature T=1.3 K as a pronounced minimum of the negativity observable close to the first critical field B≈23 T, which is subsequently followed by an anomalous rise of the negativity until the external magnetic field nearly reaches a midpoint of the intermediate plateau $N_{\mathrm{QFI}}=0.5$ attributable to the quantum ferrimagnetic phase $|\mathrm{QFI}\rangle$. To complete the physical understanding, Figure 7b shows the negativity as a function of temperature for a few different values of the magnetic field applied along the crystallographic $c^{*}$-axis by assuming the same set of the interaction parameters. It is quite clear that zero-temperature asymptotic limits of the negativity are fully consistent with aforedescribed field dependencies. Besides, the inset of Figure 7b displays an energy gap between a ground state and a first excited state as a function of the magnetic field, which in turn offers a simple explanation why the negativity of size $N_{\mathrm{QAF}}\approx 1$ and $N_{\mathrm{QFI}}=0.5$ are most robust against rising temperature at the magnetic field B=0 T and 28 T, respectively. As a matter of fact, the largest energy gap of the quantum antiferromagnetic ground state $|\mathrm{QAF}\rangle$ is at zero field and the one of the quantum ferrimagnetic ground state $|\mathrm{QFI}\rangle$ is roughly around 28 T. Finally, it is worth mentioning that the negativity may show a peculiar temperature-induced rise when the external magnetic field exceeds the saturation field (see for instance the dependence for B=35 T). Under this condition, the negativity starts from zero due to presence of the classical ferromagnetic ground state $|\mathrm{FM}\rangle$ at zero temperature, but a thermal population of a few low-lying excited quantum states may give rise to a relatively weak thermal entanglement.

Next, let us turn our attention to the most essential features of the negativity of the NAOC complex when the external magnetic field is applied along the crystallographic *a*-axis. To this end, Figure 7c,d involve analogous magnetic-field and temperature dependencies of the negativity, which were calculated for the spin-1 Heisenberg dimer unambiguously characterized by the second reported fitting set of the parameters inherent to this specific field orientation. Figure 7c shows the negativity as a function of the magnetic field for a few different temperatures and Figure 7d shows the negativity as a function of temperature for a few different values of the magnetic field. Although qualitatively the same patterns in the respective field and temperature dependencies can be recognized as in Figure 7a,b, there is an enormous quantitative difference in a size of the field range corresponding to an intermediate plateau of the negativity $N_{\mathrm{QFI}}=0.5$. This value can be naturally ascribed to the quantum ferrimagnetic phase $|\mathrm{QFI}\rangle$, which appears in much wider magnetic-field range from B=20 T until B=44 T. It is quite obvious that the quantum ferrimagnetic phase $|\mathrm{QFI}\rangle$ is crucially stabilized by the easy-plane single-ion anisotropy $D_{a}/k_{B}=4.91$ K, which simultaneously makes the crystallographic *a*-axis a hard magnetization axis. From a detailed analysis of the energy gap, which is shown in the inset of Figure 7d, one may conclude that the initial value of the negativity $N_{\mathrm{QFI}}=0.5$ ascribed to the quantum ferrimagnetic phase $|\mathrm{QFI}\rangle$ is the most robust against rising temperature at the particular value of the magnetic field B≈32T that coincides with the largest energy gap. The largest resistance of the negativity $N_{\mathrm{QAF}}\approx 1$ pertinent to the quantum antiferromagnetic phase $|QAF\rangle$ against temperature-driven decline can be repeatedly observed at zero magnetic field in agreement with the largest energy gap of the $|QAF\rangle$ ground state.

**Figure 8:**
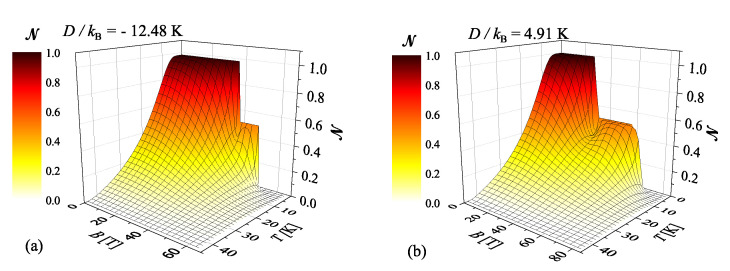
The negativity of the NAOC complex in the form of 3D plot versus temperature and magnetic field. The relevant theoretical predictions are based on the spin-1 Heisenberg dimer (1) with the isotropic coupling constant $J/k_{B}=30.66$ K and Δ=1 by considering two different spatial orientations of the magnetic field applied either along to the crystallographic $c^{*}$- or *a*-axis: (**a**) $D_{c^{*}}/k_{B}=-12.48$ K, $g_{c^{*}}=2.28$; (**b**) $D_{a}/k_{B}=4.91$ K, $g_{a}=2.24$.

Finally, 3D plots of the negativity of the NAOC complex versus temperature and magnetic field are shown in Figure 8 for two different spatial orientations of the magnetic field applied either along the crystallographic $c^{*}$- or *a*-axis. The relevant 3D plots can be regarded as a certain type of the phase diagram, which allocates the parameter space with or without thermal entanglement. It is worthwhile to remark that the strong enough thermal entanglement of the NAOC complex persists up to T≈40 K irrespective of a spatial orientation of the external magnetic field. On the other hand, the persistence of the thermal entanglement against the external magnetic field strongly depends on its spatial orientation due to a substantial size of the uniaxial single-ion anisotropy. If the magnetic field is applied along the easy magnetization axis identified with the crystallographic $c^{*}$-axis, the sizable thermal entanglement survives nearly up to a relatively high magnetic field B≈34 T comparable with a size of the exchange-coupling constant. Contrary to this, the magnetic field applied along the crystallographic *a*-axis being the hard magnetization axis for the NAOC complex has much more gentle effect upon suppression of the thermal entanglement, which is accordingly maintained up to much stronger magnetic fields B≈44 T. Bearing all this in mind, it could be concluded that the NAOC compound shows sufficiently strong thermal entanglement up to relatively high magnetic fields, which can be additionally enhanced or lowered by a proper choice of the spatial orientation of the applied magnetic field. On the other hand, the persistence of the thermal entanglement of the NAOC complex against rising temperature holds up to T≈40 K and is not affected anyhow by a spatial orientation of the magnetic field.

## 5. Conclusions

In the present paper, we have exactly calculated the negativity within pure and mixed states of a spin-1 Heisenberg dimer with the uniaxial single-ion and exchange anisotropies in a presence of the external magnetic field. The negativity, which may serve as a measure of bipartite entanglement at zero as well as nonzero temperatures, was rigorously calculated from negative eigenvalues of a partially transposed density matrix according to the definition put forward by Vidal and Werner [34]. In particular, we have examined in detail the negativity as a function of temperature and magnetic field for specific choices of the exchange and uniaxial single-ion anisotropies. It has been shown that the negativity shows at absolute zero temperature a stepwise dependence on the magnetic field with two sizable discontinuous jumps and intermediate plateaus at $N_{\mathrm{QAF}}\approx 1$ and $N_{\mathrm{QFI}}=0.5$ ascribed to the quantum antiferromagnetic phase $|\mathrm{QAF}\rangle$ and the quantum ferrimagnetic phase $|\mathrm{QFI}\rangle$, respectively. The discontinuous zero-temperature changes of the negativity are also clearly manifested at sufficiently low temperatures as steep but continuous magnetic-field variations of the negativity, whereas rising temperature gradually smears out the marked field dependence of the negativity. The magnetic-field-driven phase transition between the $|\mathrm{QAF}\rangle$ and $|\mathrm{QFI}\rangle$ ground states gives rise at low enough temperatures to a pronounced local minimum, which is subsequently followed by a peculiar field-induced rise of the negativity observable in a range of moderately strong magnetic fields. Another outstanding finding concerns with a theoretical prediction of a singular point-kink, which emerges in the temperature dependence of the negativity when considering the uniaxial single-ion anisotropy of easy-axis type. This peculiar phenomenon was explained in terms of a temperature-driven sign change of one eigenvalue of a partially transposed density matrix, which contributes to the negativity just below temperature corresponding to this singular point. Moreover, it has been verified that the persistence of the thermal entanglement against rising temperature strongly relates to an energy gap between a ground state and a first excited state. The negativity $N_{\mathrm{QAF}}\approx 1$ ascribed to the quantum antiferromagnetic phase $|\mathrm{QAF}\rangle$ is thus retained over the widest temperature range at zero magnetic field, while the value $N_{\mathrm{QFI}}=0.5$ pertinent to the quantum ferrimagnetic phase $|\mathrm{QFI}\rangle$ is kept constant in the widest temperature interval for the specific value of the magnetic field that nearly coincides with a midpoint of the relevant plateau.

Last but not least, the concept of negativity elaborated in the present work for the spin-1 Heisenberg dimer was also specifically adapted to the homodinuclear nickel coordination compound NAOC [48]. To bring insight into the thermal entanglement between two exchange-coupled spin-1 Ni${}^{2+}$ magnetic ions we took advantage of the fitting set of parameters reported for the NAOC complex in our previous work dealing with its magnetic properties (in particular magnetization and susceptibility) [51]. It turns out that the resistance of the bipartite entanglement of the NAOC complex against the magnetic field depends basically on a spatial orientation of the magnetic field due to a sizable uniaxial single-ion anisotropy. On the contrary, the robustness of the bipartite entanglement of the NAOC complex against rising temperature is not magnetic-field-orientation dependent when it persists up to nearly the same temperature T≈40 K regardless of a spatial orientation of the external magnetic field. The technological applications in modern quantum computation and quantum processing of information would however require persistence of the thermal entanglement up to much higher temperatures.

Among the large family of homodinuclear nickel complexes bridged through the oxalate group, the coordination nickel compound [Ni${}_{2}$(cyclam)${}_{2}$ox](NO${}_{3}$)${}_{2}$ (ox = oxalate and cyclam = 1,4,8,11-tetraazacyclotetradecan) [53] represents most promising candidate for a stabilization of the thermal entanglement to higher temperatures, because the relevant coupling constant $J/k_{B}=56$ K is sufficiently high in order to stabilize the thermal entanglement approximately up to T≈70 K. A greater stabilization of the thermal entanglement could be reached by substituting the bridging oxalate group through azido bridges, because the azido bridges may transmit superexchange coupling much more effectively in comparison with the oxalate group. Indeed, the homodinuclear nickel complex [Ni(dl-cth)($\mu_{1,3}$-N${}_{3}$)]${}_{2}$(ClO${}_{4}$)${}_{2}$ (dl-cth = 5,5,7,12,12,14-hexamethyltetraazacyclotetradecan) [54] has a much stronger value of the coupling constant $J/k_{B}=161$ K, which indicates the existence of nonnegligible thermal entanglement up to relatively high temperatures T≈200 K.

## Data Availability

The data presented in this study are available on request from the corresponding author.

## Abbreviations

The following abbreviations are used in this manuscript:

ESR	Electron spin resonance
NAOC	[Ni${}_{2}$(Medpt)${}_{2}$(μ-ox)(H${}_{2}$O)${}_{2}$](ClO${}_{4}$)${}_{2}$·2H${}_{2}$O
Medpt	3,3${}^{\prime }$-diamino-N-methyl-dipropylamine
ox	oxalate
cyclam	1,4,8,11-tetraazacyclotetradecan
dl-cth	5,5,7,12,12,14-hexamethyltetraazacyclotetradecan

## Appendix A

Explicit form of eigenvectors and eigenvalues of Hamiltonian 1 are as follows:

$$\begin{array}{cc} |\psi_{1}\rangle & =\frac{1}{2}\left[A_{+}\left(|1,-1\rangle +|-1,1\rangle \right)-\sqrt{2}A_{-}|0,0\rangle \right],\;E_{1}=-\frac{1}{2}J+D-R\\ |\psi_{2}\rangle & =\frac{1}{\sqrt{2}}\left(|1,-1\rangle -|-1,1\rangle \right),\;\;E_{2}=-J+2D\\ |\psi_{3}\rangle & =\frac{1}{\sqrt{2}}\left(|1,0\rangle -|0,1\rangle \right),\;\;E_{3}=-J\Delta +D-h\\ |\psi_{4}\rangle & =\frac{1}{\sqrt{2}}\left(|-1,0\rangle -|0,-1\rangle \right),\;\;E_{4}=-J\Delta +D+h\\ |\psi_{5}\rangle & =\frac{1}{2}\left[A_{-}\left(|1,-1\rangle +|-1,1\rangle \right)+\sqrt{2}A_{+}|0,0\rangle \right],\;E_{5}=-\frac{1}{2}J+D+R\\ |\psi_{6}\rangle & =\frac{1}{\sqrt{2}}\left(|1,0\rangle +|0,1\rangle \right),\;\;E_{6}=J\Delta +D-h\\ |\psi_{7}\rangle & =\frac{1}{\sqrt{2}}\left(|-1,0\rangle +|0,-1\rangle \right),\;\;E_{7}=J\Delta +D+h\\ |\psi_{8}\rangle & =|1,1\rangle ,\;\;E_{8}=J+2D-2h\\ |\psi_{9}\rangle & =|-1,-1\rangle ,\;\;E_{9}=J+2D+2h \end{array}$$

in which *R*, $A_{+}$ and $A_{-}$ are defined as:

$$R=\sqrt{{\left(\frac{J}{2}-D\right)}^{2}+2{\left(J\Delta \right)}^{2}},\;\;A_{\pm }=\sqrt{\frac{R\pm (\frac{J}{2}-D)}{R}}.$$

The partition function is given by:

$$\begin{array}{c} Z=\sum_{i=1}^{9}\exp\left(-\beta E_{i}\right)=\exp\left[\beta \left(J-2D\right)\right]+2\exp\left[-\beta \left(J+2D\right)\right]\cosh\left(2\beta h\right)\\ +4\exp\left(-\beta D\right)\cosh\left(\beta h\right)\cosh\left(\beta J\Delta \right)+2\exp\left[\beta \left(\frac{J}{2}-D\right)\right]\cosh\left(\beta R\right). \end{array}$$

## Appendix B

Explicit form of nonzero elements of the density matrix are as follows:

$$\begin{array}{cc} \rho_{11} & =\frac{1}{Z}\exp\left[-\beta \left(J+2D-2h\right)\right]\\ \rho_{22} & =\frac{1}{Z}\exp\left[-\beta \left(D-h\right)\right]\cosh\left(\beta J\Delta \right)\\ \rho_{33} & =\rho_{77}=\frac{1}{2Z}\exp\left[-\beta \left(D-\frac{J}{2}\right)\right]\left\{\cosh\left(\beta R\right)+\frac{\frac{J}{2}-D}{R}\sinh\left(\beta R\right)+\exp\left[-\beta \left(D-\frac{J}{2}\right)\right]\right\}\\ \rho_{44} & =\frac{1}{Z}\exp\left[-\beta \left(D-h\right)\right]\cosh\left(\beta J\Delta \right)\\ \rho_{55} & =\frac{1}{Z}\exp\left[-\beta \left(D-\frac{J}{2}\right)\right]\left\{\cosh\left(\beta R\right)-\frac{\frac{J}{2}-D}{R}\sinh\left(\beta R\right)\right\}\\ \rho_{66} & =\frac{1}{Z}\exp\left[-\beta \left(D+h\right)\cosh\left(\beta J\Delta \right)\right]\\ \rho_{88} & =\frac{1}{Z}\exp\left[-\beta \left(D+h\right)\right]\cosh\left(\beta J\Delta \right)\\ \rho_{99} & =\frac{1}{Z}\exp\left[-\beta \left(J+2D+2h\right)\right]\\ \rho_{24} & =\rho_{42}=-\frac{1}{Z}\exp\left[-\beta \left(D-h\right)\right]\sinh\left(\beta J\Delta \right)\\ \rho_{35} & =\rho_{53}=\rho_{57}=\rho_{75}=-\frac{1}{\sqrt{2}Z}\sqrt{1-{\left(\frac{D-\frac{J}{2}}{R}\right)}^{2}}\exp\left[-\beta \left(D-\frac{J}{2}\right)\right]\sinh\left(\beta R\right)\\ \rho_{37} & =\rho_{73}=\frac{1}{2Z}\exp\left[-\beta \left(D-\frac{J}{2}\right)\right]\left\{\cosh\left(\beta R\right)+\frac{\frac{J}{2}-D}{R}\sinh\left(\beta R\right)-\exp\left[-\beta \left(D-\frac{J}{2}\right)\right]\right\}\\ \rho_{68} & =\rho_{86}=-\frac{1}{Z}\exp\left[-\beta \left(D+h\right)\right]\sinh\left(\beta J\Delta \right) \end{array}$$
